# Gynæcological Society of Chicago

**Published:** 1889-01

**Authors:** 


### GYNÆCOLOGICAL SOCIETY OF CHICAGO.

*Regular Meeting, Friday, November 16th, 1888.*

The President, Charles T. Parkes, M.D., in the Chair.

Professor A. Reeves Jackson read a paper on

### SOME UNCURED CASES OF UTERINE HÆMORRHAGE.

I desire to report the histories of some of the cases in which I have failed to cure a rather frequent ailment—uterine hæmorrhage.

*Case I.*—Mrs. F. W. first consulted me on July 7th, 1884. She was 28 years old, and had been married two and a half years; no pregnancy. Menstruation commenced at the age of sixteen, and had always been regular and in every way normal down to the time of marriage. After that event, a period occurred at proper time. The patient then missed two periods. After lifting a heavy weight, a flow of blood appeared and continued for several weeks. She visited Dr. Wm. Goodell, of Philadelphia, who curetted the uterus in May, 1883. I do not know what, or whether anything, was removed, but the patient considered herself well for four months. In the following autumn, two periods were again missed. After a trifling misstep a red flow began, and continued about one-half of the time for nine months; then I saw her. She was a large but not tall woman, weighing one hundred and sixty-two pounds, of dark and rather dull complexion, and habitually despondent temperament. There was no history or present evidences of any disease of important thoracic or abdominal organs. The pelvic viscera were found to be entirely normal in size and position. The os uteri was rather small, but of virginal shape, and free from redness or erosion.

On July 9th, I administered ether, and curetted the uterine cavity. A few fungous granulations were removed, and an application made of Churchill's iodine solution. This latter was repeated every four to seven days. For about three months menstruation appeared at intervals of four or five weeks, rather profusely, and lasting from seven to ten days. Then the inter-hæmorrhagic periods became shorter —about three weeks. During the spring of 1885, the intervals were about two weeks; once only nine days intervened. In the early part of 1886, the flow was more irregular, recurring every three to six weeks. In July, however, it appeared after an interval of two weeks and lasted three weeks, not profusely but constantly. In August following, I again used the curette, without any result. Nothing was removed, and no change was produced in the symptoms. In June, 1887, the same operation was followed by a like negative result. A month later, my connection with the case ceased. During the time of my attendance (which was not continuous, but interrupted sometimes for periods of several months) the treatment consisted in efforts to overcome habitual constipation, and the intra-uterine application of iodine, carbolic acid, a solution of ferric alum, etc. These applications were always preceded by the introduction of a No. 12 bougie. There were two unfavorable conditions present in this case which I could not obviate. The one was tight-waist dressing, and the other an insuperable objection to active exercise.

Within the past few days the husband of this patient called upon me and stated that the subsequent history of his wife presented no material change; that, at times, she seemed better, and then became worse again. She is now under the care of a prominent physician of this city, and is thought to be improving.

*Case II.*—Mrs. E. D. placed herself under my care January 22d, 1885. She was a Jewess, had been married three years, and was never pregnant. Menstruation began at twelve, had never been distinctly regular, and for many years was rather scanty. During the past year, however, the discharge had recurred with greater frequency than ever before, the interval being less than four weeks, and it had become shorter and shorter, while, at the same time, the quantity of discharge at each period had steadily increased. During the month immediately preceding my first interview with the patient, there had been three attacks of flowing. There was no evidence whatever of cardiac, pulmonary, hepatic, or renal disorder. The patient ate well, slept well, and felt well. She was only annoyed by the frequency of the bloody flow. A pelvic examination revealed no abnormal condition, position, or shape of any of the organs. The single disordered symptom discoverable consisted in a discharge of dark blood which was seen slowly oozing from the os uteri. The introduction of the sound was easily accomplished and was followed by a rather profuse flow of redder blood.

Two days later, under ether anæsthesia, the cervical canal was moderately dilated, and the interior of the uterus curetted. A small number of fungosities were removed and the uterine cavity swabbed with Churchill's iodine solution. On February 20th, a bloody discharge appeared, and continued more or less profusely until March 14th—three weeks. I then made an application of Monsel's solution of iron. The discharge ceased for two days; it then reappeared and lasted to March 23d—seven days. I again dilated the cervical canal and passed in a curette, without removing anything, and applied the iodine solution. There was no discharge for eight days subsequently. Then a flow which seemed like that of menstruation appeared. It lasted five days, ceased two days, reappeared and continued with occasional intervals of one or two days until April 23d, when it became profuse and seemed like menstruation again. After a few days the flow diminished, but a reddish-tinged oozing continued until a more profuse flow indicated the return of catamenial discharge. On November 9th, the patient reported that the longest time she had been free from bloody discharge was four days. During all that time, I had availed myself of frequent opportunities of making local applications of iodine, carbolic acid, Monsel's solution, alum, fused nitrate of silver, etc. Also, I had given quinine, ergot, and viburnum, and on several occasions I had gently dilated the cervical canal, and tried to get away something with the dull curette.

No especial change was noticed in the symptoms in the summer of 1886, during which time all treatment was suspended. In January, 1887, the patient informed me that once only she was free from hæmorrhage for a period of three weeks. Treatment was then resumed. I introduced a tupelo tent and followed the dilatation by a very thorough application of iodized phenol. This was repeated at intervals of from five to seven days for several weeks. There was no improvement. Then I tried nitrate of silver for awhile, but the hæmorrhage continued. In the summer of 1887, the patient went to Europe with her husband, and for a few weeks while there she was somewhat better. In the following autumn, however, after her return, the hæmorrhage was as constant as before. On December 23d, I dilated the uterine canal mechanically under anæsthesia, and removed two large granulations. She died one week later of peritonitis.

*Case III.*—Mrs. W. L., aged 35 years, had been married seven years, and had two children, the younger being sixteen months old. I saw her on September 5th, 1885. When the baby was ten months old, menstruation appeared, and for two periods was quite normal, lasting four days. The third came a week too soon and lasted two weeks. After an interval of two weeks it again appeared, and when it had persisted ten days, necessitating two or more napkins daily, she applied for relief. She then showed effects of the loss in pallor, impaired strength, and enfeebled digestion. The baby was weaned, and the patient took strychnia, quinine, and arsenic. I found no pelvic cause for the hæmorrhage unless it might have been a slight degree of subinvolution.

The uterus was freely movable, in normal position, free from erosion, and there was no swelling or undue tenderness in the region of the ovaries, tubes, or broad ligaments. Constipation was present, and I prescribed salines and a regulated nutritious diet. Subsequently, the hæmorrhage continuing more or less constantly, although not profusely, other means were tried, many of them rather empirically. They included ergot, hydrastis, viburnum—all without perceptible effect. Then I dilated the cervical canal and explored the interior of the uterus with finger and curette. Nothing was found.

After about eight months, the patient placed herself under the care of an electrician, who treated her for nearly six months. Then she went to Cincinnati, and remained several months under the care of a distinguished gynæcologist. She returned to me in January last, and stated that she had not at any time been more than ten days without a bloody discharge, and rarely more than three days. The menstrual periods were distinguishable by the more profuse flow which marked their presence, and they were fairly regular.

Once more I dilated the cervix under etherization, and drew a curette over every part of the intra-uterine surface, taking especial care to get the instrument into the cornual depressions. No tissue was brought away, and very little blood. I subsequently made internal applications of tinct. iodine, tannin, Monsel's solution, and once, at the close of what seemed to be a menstrual period, I applied nitric acid. While, at times, there was a diminution of the discharge, the improvement was only temporary, and in July last she again passed from my care.

*Case IV.*—The notes of the following case were kindly furnished me by Dr. J. H. Stowell. M. M., a single woman, 25 years of age, was born in Canada. Menstruation commenced at twelve, and was quite regular until she was nineteen; then the flow became prolonged, lasting sometimes five or six weeks. This was followed by regularity, both in quantity and times of recurrence for a few months, when menorrhagia again appeared. At the age of twenty-two she was examined by a physician at Ottawa, who curetted the uterus, with what immediate result is not known, but the patient was free from all sanguineous discharge during a period of three months. Menstruation then returned and was regular and in every respect normal for the following four months. Becoming menorrhagic again the curetting was repeated, with the same temporarily beneficial results as before. Again after a few months, the flow became profuse and a third curetting was made six months after the second, the latter time without apparent benefit.

The patient removed to this city and came under the care of Dr. Stowell, with whom I saw her in the early part of the current year. On January 21st, after thorough dilatation of the uterine canal, I curetted very carefully, removing a small number of granulations. The operation was followed by the application of Churchill's iodine. For two months subsequently there was no bloody discharge. Then it reappeared, and Dr. Stowell informs me that he has continued to treat the symptom in a routine manner locally and generally, but without satisfactory results. Latterly this case presents a curious feature: the flow is very profuse every alternate month, and normal the other.

It will be observed that in the foregoing cases there were some features common to them all. Thus, in none of them did the hæmorrhage take the form of rapid, profuse flow suddenly exhausting the patient, as we not infrequently notice in cases in which the discharge depends upon an abortion, or upon the presence of cancer or fibroid neoplasm. On the contrary, the evil effects produced were the result rather of the persistence of the discharge covering long periods of time. Again, in none of them was there evidence of disease of the lungs, heart, liver, kidneys, or other important organs causing a depraved condition of the blood or pelvic plethora. In none of them was there a history of hematocele, pelvic inflammation, or detectable pelvic swelling. In none of them was there any displacement or flexion of the uterus, or dislocation of the ovary. Indeed, in none of them could I determine a sufficient local or systematic cause. I have thought that possibly some such cause might exist in disordered condition of the Fallopian tubes or pelvic cellular tissue not ascertainable by our present known methods of investigation.

Being aware of the influence of malarial poison in producing congestion of the abdominal and pelvic viscera, including perhaps the mucous membranes, I have not failed to take this possible etiological factor into consideration and to make careful inquiry with the view of ascertaining its existence. The result in each case was wholly negative.

Dr. D. T. Nelson: I cannot say that I have seen just such cases; I only regret that the doctor was not able to follow the cases even longer. One died; I would like to know if there was any post-mortem examination. Perhaps the cause of death was such that it would hardly be satisfactory if there were a post-mortem. It seems to me that the only way we can arrive at a conclusion as to the nature of the disease and consequently the proper method of treatment, is to follow them longer. One case that I have seen, I was fortunate enough to see one or two years after a somewhat similar experience by other gentlemen, in which nothing was reported to have been found, in which there was curetting; but as I saw it subsequently there was plain evidence of fibroid tumor. Perhaps if these cases were seen longer or examined post-mortem there would be a satisfactory explanation of the persistence of the hæmorrhage. I think one of the small single or multiple fibroid tumors, which cannot be detected by the most skillful finger, or by any means of examination that we have at present, are likely to produce such results, and I believe they may lie dormant for a longer period than these cases have been observed; I am quite sure that I have seen them—and eventually, perhaps of their own nature, develop so as to be found; perhaps from some exciting cause other than that. There is another cause, it seems to me, for these hæmorrhages, concerning which I can hardly speak rightly: Will disease of the Fallopian tubes produce hæmorrhage from the uterus? I do not mean by that a salpingitis that will fill the tube with pus or serum, and be so large as to be easily felt, but actual disease of the mucous membrane, perhaps not extending much deeper than the mucous membrane, but capable of producing a continuous hæmorrhage. I simply throw this out as a hint, as a possible explanation, for further examination. Perhaps it is not a parallel case, and yet it seems to me that there is a similar cause. A woman after menstruation, if you please, a week after the cessation of the menstrual period, takes cold, has a slight cough, has a traumatism or some slight disturbance in the pelvis giving pain and other evidences of inflammation; we all know that one of the most common symptoms attending upon the pain, etc., is hæmorrhage from the uterus. Not a severe one, perhaps, but a slight flow. You may say that means congestion of the whole pelvic viscera. True, it ordinarily does. But what does produce the uterine flow? Is it the uterus alone, is it the ovaries alone, is it the broad ligaments only, is it the Fallopian tubes and the uterus, is it the Fallopian tubes only? It does seem to me in some instances that it is not only the uterus, not only the broad ligaments and vascular structures about the uterus; but that the Fallopian tubes and ovaries have very much to do with this condition. I believe if these cases were observed long enough, we should find a congestion of the uterus produced by a fibroma or sarcoma, or disease of the Fallopian tubes or ovaries, and pre-existing disease of the ovaries and tubes produce hyperæmia of the uterus that favors the development of fibroma and sarcoma.

Professor C. T. Parkes: A case was recently reported in one of the foreign journals where all these remedies were used; curetting and intra-uterine dilatation, and finally the surgeon concluded he would make an abdominal section. After making the section, he found what he reports as a cavernous angioma of one of the ovaries. It was removed and the case cured.

Dr. Robert Tilley: It is my special interest in the last case reported by Dr. Jackson that has given me the honor of being present this evening. The reason why the patient came more or less under my observation was this: A distant relative, who is an intimate friend of mine, had been instrumental in bringing the patient here from Canada, and was a good deal concerned about the fact that she was not any better. She came to me for general counsel as to what would be the best course for her to adopt under the circumstances. I advised her to get letters from the doctors who had been in attendance on her and to send them to her parents, with the supposition that they would consult with the family physician who had previously curetted the uterus. Meanwhile, the patient was flowing considerably, and she asked me if I would not give her such general instructions as would enable her, at any rate, to be freed from a certain amount of anxiety which she necessarily would be under apart from that. I said that under those conditions I would, with the understanding that if any serious symptoms developed I should not think of taking charge of the case. I resorted to turpentine, 10-drop doses in an emulsion, and after she had taken two doses of this turpentine, there was a sensation of strangury, and it was stopped for one day. With that strangury there was a manifest diminution of the flow. I concluded that it was fair to infer that the organs in the pelvic regions were, in all probability, susceptible to the turpentine, and I recommended its use in smaller doses and added to it some camphor water. She continued to use the turpentine, and the flow continued to diminish; there was no longer any sensation of strangury after she had used the camphor water in conjunction with it, and on Saturday last I was informed that the flow had ceased altogether. She called at my office, and I found that the pulse, which had been previously 100, was reduced to 80, and the peculiar sensation of palpitation which she has complained of as existing before was almost completely removed, and she was feeling in every way a good deal better. I heard from her again on Tuesday, she was then on her way to one of the suburbs, with the understanding that I should hear from her again to-morrow. I feel satisfied that the flow has not returned, otherwise I should have heard. The use of the turpentine seems to have had the special advantages of securing, at any rate, a temporary cessation of the hæmorrhage.

Professor Jackson: These cases I have reported are not the only ones which I have failed to cure, but I had accurate notes of these, and I considered them sufficient for the purpose of bringing the matter before the society. The existence of fibroids as a probable cause in some of these cases, as suggested by Dr. Nelson, had occurred to me. The question of possible pregnancy also arose, and I made such inquiries and investigations as I hoped would lead to a settlement of the latter point especially. We know that in fibroid growths of the uterus hæmorrhage becomes by-and-by a prominent symptom, and usually it is the one which first calls the attention of the patient to her condition; and in these cases we usually have no trouble in the diagnosis where there is present either a polypus or a fibroid of sufficient size to alter the shape of the uterus or to be felt within the cavity of the organ. I can conceive how a very small fibroid, incapable of detection by ordinary methods and ordinarily skilled hands and fingers, might produce hæmorrhage, but would it yield to the medical treatment of a fibroid? If it did not, we should hardly feel justified in making a laparotomy to discover the existence of a fibroid that presented no other evidence of its existence than hæmorrhage. It is true that patients nowadays submit to laparotomy when they are urged to do so; yet the one who urges should have some kind of objective reason upon which to base his advice. In none of these cases could I detect any change whatever in the shape of the uterus. The fact is that, when we find a fibroid as the cause of hæmorrhage, it is already surgical. It is large enough sometimes to have existed, with their slow rate of growth, for many years, and yet hæmorrhage has probably not been present until the latest part of the history.

Disease of the Fallopian tubes I have no doubt is a frequent cause of persistent uterine hæmorrhage. Primarily, the blood may have its source in the tube; later, by overaction—by the mere bleeding—there is produced manifest disease of the endometrium, as in the case last cited. But, granting this, inasmuch as we can only reach the uterine lining anyhow by our topical treatment, even if we have a suspicion that there may be tubal disease, it does not aid us in deciding what to do for it. The ethics of the condition are in doubt.

Allusion has been made to the death of the third patient. She died of peritonitis following the operation. I have reported it in order to call attention to the danger of what is usually considered a simple and safe operation. In this case, the operation was done under the strictest antiseptic precautions; every care was taken to avoid any danger from that source. Unfortunately, I was obliged to leave the city shortly after the operation, leaving the patient in an unsatisfactory condition, temperature 102°, rapid pulse, red cheeks, and evident high constitutional excitement. On my return I found her still worse, and shortly afterwards she died of peritonitis, whether septic or not I do not know. The dilatation was mechanical; was not made by tents, which I consider a dangerous method of treatment.

I did not mention in the paper an important fact in the subsequent history of the last case. It was this: During the last examination I made, I detected a distinct swelling at the side of the uterus, apparently in the Fallopian tube. Then I suggested that if the patient did not get better, here was a reason why we might properly make a laparotomy—not for the hæmorrhage, but for the swelling which was possibly the cause of it. The curetting on three occasions certainly had a very satisfactory effect. On one occasion, for four months subsequent to the operation, the patient had no bloody discharge whatever, natural or unnatural, which would seem to indicate that, at that time at least, the cause of the hæmorrhage was in the uterus, and that the effect of the remedies applied more thoroughly after the dilatation had a controlling influence for a long time. This is very satisfactory to a patient who has been having a profuse or prolonged hæmorrhage. This patient was under the care of excellent men in Canada, who, so far as I know, never detected any swelling, and I infer from this fact that the latter symptoms were of comparatively recent appearance.

The nitric acid, which I applied in one case, I formerly used a great deal more than I do now. My method of using it is that recommended by Athill some years ago. The uterus is previously dilated, and when sufficiently open, the pure nitric acid is passed into the uterine cavity on an ordinary cotton-wrapped applicator. I place the patient on her back, introduce a perineal depressor, seize the anterior lip of the uterus, draw the organ down, and surround it entirely with cotton which has been dipped in a solution of bicarbonate of soda. The applicator is dipped into the acid, passed into the uterine cavity, and held there for a few minutes. It is then withdrawn and the vagina syringed until all fuming ceases. Then I remove the tampons from about the cervix, and another tampon saturated in glycerin is placed over the os uteri. Such was the method used in this case. The nitrate of silver I applied in this way: A few grains of the crystal are melted in the bottom of a test tube. A silver uterine probe is dipped into the fluid and withdrawn a few times, until a bead is formed on the end of the instrument the size of a grain of rice. This is passed through a speculum up into the interior of the uterus, and held there for a few minutes, and slowly drawn out so as to come in contact with the surrounding membrane. This method has been recommended as very effective in chronic cases of menorrhagia. The hydrastis I have used a good deal. Loewenthal has reported several cases in which he had used hydrastis for preventing menstruation absolutely in persons to whom it was injurious; chlorotid and anæmic patients, he affirms, have been effectually cured by the use of hydrastis, given so as to absolutely obliterate the function of menstruation.

Ergot I have not found to be beneficial in any condition of the uterus involving hæmorrhage, unless it be subinvolution during its soft stage where the uterus is large and heavy. In those cases I believe ergot is an excellent remedy. That condition was not present in any of these cases. Whether in one of them the cessation of menstruation for two months indicated pregnancy, I do not know, but there was no other evidence of it; there was no fœtus observed; nor was the history that of an early abortion, so that the single symptom of absent menstruation in a woman who was irregular at times was not so significant as it might be under other circumstances.

Dr. Addison H. Foster read a paper entitled

A CASE OF HYDRAMNION.

December 11th, 1875.—Mrs. W. English, aged 27, was taken in premature labor at the eighth month with her third child. On my reaching the case, the abdomen was found enormously distended, the pains regular but feeble; the os fully dilated; the membranes intact but firm; the presentation doubtful. In the interval of pains the finger was carried high up as possible anteriorly upon and through the sac, and held there to retard the discharge of the fluid, in order to prevent prolapse of the cord and sudden emptying of the uterus, and to lessen the danger of hæmorrhage.

The patient being upon an oil-cloth, scantily covered, a large empty tobacco pail, holding fifteen quarts, was seized and held under the overhanging cloth, until it was filled brimfull with normal appearing amniotic fluid; some fluid was lost in the bed and on the floor. With three or four pains, a small well-formed but immature child was expelled, that lived but a few hours.

The placenta was adherent on one side, requiring forcible digital separation from its site. It was small, being thin and fibrous on the adherent side. There was no unusual hæmorrhage following, as the uterus contracted firmly and remained so. The patient made quite a prompt and normal recovery.

There had been very little œdema of the limbs and no history of suffering from unpleasant symptoms, excepting from the distention which came on principally the last two months. Her former labors had been normal. There was no history of any *specific* disease.

I made no test or examination of the fluid or microscopical investigations of the placenta, as would now be done, with an awakened interest in the various placental phenomena met with. Hence, I am not prepared to demonstrate the causes in this case, but I wish simply to place it on record as one more contribution of its kind.

Authorities are not well agreed upon the etiology of this disease. Most authorities regard hydramnion as a disease of the fœtus or its membranes, and look there for its cause. Landois and Stirling state that "in the pathological condition of hydramnion, the blood-vessels of the uterine mucous membrane secrete a watery fluid" (p. 882, 2d ed.). According to Charpentier, one of these three (3) kinds of lesions are usually present: "1st. Those of a supposedly inflammatory nature. 2d. Fœtal malformations. 3d. Lesions of the uterus, or tumors of various kinds. Among the inflammatory are found degenerations and adhesions of the placenta, to which category our case evidently belongs. The same author states that the following facts have been proved by various observations:

1st. Dropsy of the amnion coincides very frequently with twin pregnancy.

2d. That children born with the complication of dropsy of the amnion are often the subjects of malformations and monstrosities.

3d. That a certain number of these cases are syphilitic.

4th. Hydramnion is very rare in primiparæ and comparatively rare in second pregnancies.

This case developed it at her third pregnancy.

From the cases on record, the amount of fluid that has occasioned trouble varied from ten to sixty pounds, as a rule, though five pounds have occasionally done so. In the case I have cited the amount could not be far from forty-five pounds.

In regard to the prognosis, that for the fœtus is always very serious. For the mother, not so serious, except in acute hydramnion, although the danger of uterine inertia and hæmorrhage in all cases is considerable,

Dr. Sawyer: The senior Dr. Byford, in the early history of this Society, called attention to the fact that hydramnios was frequently associated with monsters or the acephalous fœtus. That statement called to mind that I had seen two or three cases of spina bifida that contained enormous quantities of fluid, and it occurred to me then that the arachnoid membrane, which is open in the acephalous fœtus, is capable of secreting an enormous amount of fluid. Is it impossible that the fœtus does really contribute most of this fluid? I have seen one case of hydramnion with a woman pregnant for the third time.

I remember one case--the one I have already alluded to—in which labor that I thought was surely begun was greatly interfered with by the feebleness of the uterus, due to its great distention, and in that case I introduced a catheter through the cervix high up and drained off all that I thought necessary, withdrew the catheter and there was no further drain, and the labor progressed rapidly and favorably to the injury of neither mother nor child. It is a simple thing to put a male catheter, when the os is patulous, as high up as you please, and rupture the membrane and tap the amniotic sac high up.

Dr. A. H. Foster: In regard to the treatment of this case, I attended her in her second pregnancy, and was not aware of her third until I was called and found her in the condition I did, as she did not expect to have to call me for another month, so I had to act at once. I should think that the idea of aspiration would be a good one. If I should be called to a case early enough and find these conditions, I should be very happy to have Dr. Earle with me to try the experiment.

Dr. Edward B. Weston read a paper entitled

### A NEW PROCEDURE IN CASES OF ANTICIPATED COMPLETE RUPTURE OF THE PERINEUM.

On October 4, 1888, I was, for the fourth time, called to attend Mrs. H. in labor. She is a woman somewhat below the average size, and has rather a narrow pelvis, while her children are all large at birth. At the birth of her first child, a boy who weighed twelve pounds, she received a complete laceration of the perineum. The second child, also a boy, weighed nine and one-half pounds, and the perineum was torn to the anal sphincter. The third pregnancy was terminated in the sixth month by unknown cause. The child was of course small, but delivery took place very rapidly, and there was again a rupture, though not to the same degree as in the second labor.

On visiting the patient at the beginning of her last labor, an examination showed a well-restored perineum, and a child seemingly very large presenting in the first position.

Meditating over the situation, remembering what had taken place in her previous labors, I feared a complete rupture would again occur, however well I might apply the various methods or procedures for protecting the perineum. The thought came to me that it would be well to introduce a deep suture before the laceration occurred, and before the head began to press upon the perineum, so that if complete rupture did take place I should have one suture already in place, by means of which I could easily bring the parts into accurate apposition, and which could in a measure be used as forceps or tenaculum, and be of great service in whatever after-operation might be necessary. I could see no objection to the step; so with a long curved needle I introduced a silk suture a little more than half an inch to the right of the anus, and carried it up about an inch and a half in the recto-vaginal septum, and brought it out on the left side at a point corresponding to its point of entrance. I left either end six inches long and tied them together. Again there was a laceration, though not a complete one. The child, a boy, weighed eleven pounds. Objections will, of course, be raised to the procedure which I have suggested. Let us consider a few of them.

One objection will be that we cannot tell when we are to have a complete rupture. We cannot always know in advance, and here the unexpected often happens. But in some cases we feel very sure it will occur, and it is in these cases that the stitch should be introduced. And in cases of doubt the stitch had better be placed, as it will in any event do no harm. Another objection may be that, if rupture should take place, we cannot tell what direction it will take. In the great majority of cases, externally, the laceration occurs, practically, in the median line. So that whatever its internal course may be, all the advantages claimed for the stitch will be obtained. Again: that should the procedure be recognized as proper in certain rare cases, it will be abused by too frequent use. And that, with the stitch in place and with confidence in the future assistance to be derived from it, our vigilance in protecting the perineum might be relaxed. To these objections we can only say that the conscientious accoucheur would not be influenced by them.

Some will suggest the danger of sepsis. We do not believe this need be feared. If antiseptic precautions have been observed, and the stitch buried in the tissues through its whole length, the increased danger will be nil. If, fortunately, rupture does not occur, the stitch can be cut short at one side, withdrawn, and no injury will have been done. Others will say that, even should complete rupture take place, the physician should be skillful enough to repair it by some one of the old methods. This is true; but the fact remains that there are all degrees of surgical skill, ranging from no skill to that of the highest degree.

It may also be said that the obstetrician who would introduce the stitch before laceration occurred would be the very one who would have least need of it afterwards. We grant it. But the majority would prefer to have the stitch in place, rather than to begin repairs without it, the parts being not only lacerated, but often so contused, swollen, or retracted that their normal relations can scarcely be made out.

If, by the procedure suggested, some one may be enabled to repair a complete laceration of the perineum, which otherwise would be left for a gynæcologist to repair after the patient had suffered, mentally as well as physically, for weeks or months, we think it cannot be objected to. And however expert an operator may be, it is true that the easiest, simplest way of obtaining a desired result from operative procedure is the best way.

Dr. H. P. Newman: I would suggest as a substitute for this manner of procedure one that has long been in use, namely, episiotomy, or bilateral incision of the vulva. It has served me well in one or two instances. It has this obvious advantage, that it allows of tearing laterally in such a way as not to injure the perineal muscles, or injure the recto-vaginal wall. In this way we can also prevent complete laceration, where some tearing is inevitable. I should regard Dr. Weston's method as a most admirable means of placing the initiatory stitch, where rupture seems imminent, although I cannot see how it could well obviate the tear.

Dr. E. W. Sawyer: The slight lateral incision, or episiotomy, does often prevent the laceration from occurring in the median line. I have made these incisions, one on either side, in nearly every forceps case in primiparæ, and I never saw the least harm come from it. In some instances, when I thought the perineum would be extensively torn, the laceration was really very short and insignificant. I think the suggestion of Dr. Weston is most excellent; it is in exemplification of the old axiom that a stitch in time saves nine, and I, having attempted to repair an entirely lacerated perineum more than once immediately after parturition, know something of the difficulty of introducing a deep, properly applied suture and if I could have the first suture introduced when the parts were in their normal relations, I should consider that I had overcome the greatest obstacle in immediate repair. I do not see one objection to the introduction of the suture.

Dr. Jackson: The two incisions should not be lateral but diagonal, so as to pass about one-half inch on either side of the fourchette directly outward, going beyond the rectum on either side. I have never used it, but it seems to me that in a case where complete laceration seemed inevitable it would certainly be a preferable procedure to allowing the perineum to rupture. I did not understand Dr. Weston to say that the stitch recommended by him would prevent the laceration, but that it would aid in its repair, and from this point of view I regard the suggestion as a very valuable one. It would certainly simplify the primary operation for rupture of the perineum.

Dr. E. B. Weston: I have nothing further to say, except that I did not propose, as was intimated by the first speaker, taking this stitch to prevent anything at all. It was not suggested as a preventive, but simply as an aid, in case a laceration should take place after all you had done, whether by episiotomy or any other method which you could think of, in bringing the parts together.

Dr. Bayard Holmes presented specimens of

### CULTURES OF BACTERIA FROM THE URINE OF A CASE OF NEPHRITIS AFTER SCARLET FEVER.

The specimens are of such a character that while they may not be in themselves particularly interesting, I hope they are of sufficient account, in connection with the paper I wish to present at the next meeting, to deserve a moment's attention. My paper is upon the subject of "Secondary Infection in Acute Infectious Diseases of Children." One of the diseases that I treat of is scarlet fever, and as the complication that is perhaps the most alarmimg and serious in scarlet fever is nephritis, I have taken a good deal of pains in studying the subject. Through the kindness of a neighbor physician I had a chance to examine the urine of a case of nephritis after scarlatina, and to make a few cultures from it. On the first day that any symptom of nephritis was noticed there was a very large amount of blood in the urine and I took pains to collect some of it in a perfectly sterilized flask after the first part had passed away, so as to get it in as nearly a sterile condition as possible. I took it home and planted the sediment in a few tubes of gelatin. The gelatin in a very short time showed small colonies growing along the track of the needle. From isolated colonies, the second series of tubes was planted, and they begin to show a characteristic growth. Only one of the first series of six tubes remains sterile, and only two of them begin to show any signs of mixed infection. The remaining three tubes are evidently pure cultures of the *Streptococcus pyogenes* of Rosenbach. This is the microbe that produces the enlargement of the cervical lymphatics early in scarlet fever.

